# The autonomic nervous system in atrial fibrillation—pathophysiology and non-invasive assessment

**DOI:** 10.3389/fcvm.2023.1327387

**Published:** 2024-01-04

**Authors:** Bert Vandenberk, Peter Haemers, Carlos Morillo

**Affiliations:** ^1^ Department of Cardiology, University Hospitals Leuven, Leuven, Belgium; ^2^ Department of Cardiovascular Sciences, KU Leuven, Leuven, Belgium; ^3^ Department of Cardiac Sciences, Cumming School of Medicine, Libin Cardiovascular Institute, University of Calgary, Calgary, AB, Canada

**Keywords:** atrial fibrillation, autonomic nervous system, pathophysiology, heart rate variability, heart rate turbulence, non-invasive assessment

## Abstract

The autonomic nervous system plays a crucial role in atrial fibrillation pathophysiology. Parasympathetic hyperactivity result in a shortening of the action potential duration, a reduction of the conduction wavelength, and as such facilitates reentry in the presence of triggers. Further, autonomic remodeling of atrial myocytes in AF includes progressive sympathetic hyperinnervation by increased atrial sympathetic nerve density and sympathetic atrial nerve sprouting. Knowledge on the pathophysiological process in AF, including the contribution of the autonomic nervous system, may in the near future guide personalized AF management. This review focuses on the role of the autonomic nervous system in atrial fibrillation pathophysiology and non-invasive assessment of the autonomic nervous system.

## 1 Introduction

Atrial fibrillation (AF) is the most prevalent cardiac arrhythmia and is associated with a significant burden to patients and health care (1). While clinical research on AF pathophysiology is focusing mostly on structural and electrical remodeling, there has been significantly less attention for the role of the autonomic function. Increasing knowledge on the pathophysiological process in AF, including the contribution of the autonomic nervous system (ANS) and non-invasive substrate determination, may in the near future guide personalized AF management (1). In this review, we focus on the contribution of the ANS in AF pathophysiology, non-invasive assessment of the ANS, and briefly discuss the evidence and knowledge gaps in vagally-mediated AF.

## 2 Vagal and adrenergic atrial fibrillation subtypes

In 1978 Coumel et al. described an atrial arrhythmia syndrome of vagal origin (2). They reported on 18 cases, predominantly middle-aged men without underlying heart disease, who developed progressive deterioration of AF paroxysms precipitated by vagal overactivity. They noted a particular resistance to digitalis and beta-blocking agents, while in 5 cases the arrhythmia was managed successfully by implanting an atrial pacemaker for maintaining the atrial rate (2). Historically, in prior guidelines, paroxysmal AF was classified into three types based on the involvement of the ANS (3, 4). These included vagally-mediated AF, adrenergic-mediated AF, and mixed AF. It was promptly acknowledged that the impact of such classification had limited impact on AF management (3).

The clinical characteristics of patients with vagal and adrenergic AF subtypes differ significantly. Patients with vagally-mediated AF are typically younger, more often male patients without structural heart disease and in the absence of significant cardiovascular comorbidities (5). In contrast, adrenergic AF is believed to manifest in patients by a combination of triggers and atrial remodeling due to underlying structural heart disease and risk factors such as arterial hypertension, diabetes, and heart failure (5). For example, the suggestion that nocturnal AF episodes are predominantly vagally-mediated does not take into account episodes related to obstructive sleep apnea (6). Studies reporting self-reported triggers of AF paroxysms observed that vagal triggers represented between 6% and 38% of triggers (7, 8). These studies typically limited vagal triggers to postprandial AF paroxysms, nocturnal episodes, and in the absence of any acute adrenergic trigger. Further, some patients exhibit episodes compatible with both vagal and adrenergic AF (9). A specific subgroup of patients with vagal AF are endurance athletes, where the pathophysiology of AF is hypothesized to be chronic inflammation, structural remodeling due to long periods of increased atrial pressure, and an altered sympathetic-parasympathetic balance with higher vagal tone (10). The prevalence of AF in endurance athletes have been reported to be up to 10 times higher compared to controls and are predominantly vagally-mediated (11–14).

## 3 The autonomic nervous system in atrial fibrillation

AF results from a complex interplay of triggers, substrate, and remodeling. This pathophysiological triad interacts with several anatomical structures, which can be targeted during ablation procedures.

### 3.1 Triggers

AF triggers are focal ectopic activity manifesting as atrial ectopic beats or micro-reentrant circuits (15, 16). At the cellular level, ectopic firing arises from enhanced automaticity or triggered activity. While early afterdepolarizations are related to atrial action potential prolongation with reactivation of L-type calcium channels, delayed afterdepolarizations are related to calcium overload and ryanodine receptor dysfunction of the sarcoplasmic reticulum (16). Particularly delayed afterdepolarizations are of pathophysiological importance in AF as cardiovascular diseases, such as heart failure, are associated with altered atrial calcium handling (17). Similar cellular calcium abnormalities have been identified in paroxysmal and persistent AF (18, 19). Triggered activity can result in local micro-reentry due to spatial differences in action potential duration and myofibril arrangements, such as the complexity of the muscular sleeves in the pulmonary veins (20).

In AF, adrenergic activation mediated by the sympathetic nervous system strongly promote arrhythmogenesis by boosting the calcium-dependent cardiac function and calcium-dependent triggered activity (21). Further, parasympathetic hyperactivity result in a shortening of the action potential duration by activation of the acetylcholine-activated outward potassium current (22). These acetylcholine-activated potassium channels have a higher density in the left atrium when compared to the right atrium (23). Shortening of action potential duration reduces the conduction wavelength, refractory period, and as such facilitates reentry in the presence of triggers (22, 24). In animal models, the shortening of the effective refractory period was highest in the pulmonary veins and posterior left atrium (24). This heterogeneity in response to parasympathetic nerve stimulation is attributed to both differences in nerve supply and distribution of acetylcholine-activated potassium channels (22, 25).

### 3.2 Substrate

The substrate can be defined as specific conditions that favor initiation and maintenance of AF (15). AF substrate can be divided in three types based on their etiology and eligibility for intervention. The non-modifiable substrate includes static risk factors such as age, sex, and genetic predisposition (15). The modifiable substrate are dynamic risk factors that can be targets in the management of AF with medical or lifestyle interventions, such as comorbidities as obesity, obstructive sleep apnea, and alcohol use (15). Lastly, the AF-induced substrate was introduced in 1995 as the “AF begets AF” concept as research showed that the presence of AF episodes augments the susceptibility for future AF episodes (26). In the current era this is often referred to as atrial remodeling, which are time-dependent changes of atrial myocytes and the extracellular matrix to various external stressors or risk factors resulting in persistent changes in left atrial size or function (27). Typically, the AF substrate refers to abnormalities predisposing to re-entrant circuits with areas of slow conduction prone to unilateral conduction block and altered refractoriness, such as myocardial fibrosis (28).

### 3.3 Remodeling

While structural and electrical remodeling receive most attention in research, autonomic remodeling promoting the sympathetic nervous system also plays a crucial role in AF pathophysiology. AF is associated with progressive sympathetic hyperinnervation by increased atrial sympathetic nerve density and sympathetic atrial nerve sprouting in both animal models and patients with AF (21). The coinciding adrenergic overstimulation promotes the occurrence of delayed afterdepolarization (16). Chronic adrenergic stimulation stimulates structural remodeling by increased oxidative stress, amongst others (29). This sympathetic hyperinnervation can typically be observed in patients with obesity, diabetes mellitus, heart failure, and persistent AF (30–33). However, also the extracardiac sympathetic nervous system shows clear signs of remodeling in chronic cardiovascular diseases with neuronal hypertrophy and increase in the synaptic density of the stellate ganglia (34, 35).

### 3.4 Anatomic structures

Throughout the right and left atrium, there are anatomical structures that are well-known for their predisposition to AF triggers and their specific role in the perpetuation of AF. In paroxysmal AF, up to 94% of AF triggers originate inside the pulmonary veins and may induce AF by interacting with the complexity of the myocardial sleeves at the veno-atrial junction or the local atrial substrate (36, 37). While targeting the triggers originating from the pulmonary veins is the cornerstone for patients with paroxysmal AF, in persistent and long-standing persistent AF the triggers and re-entry sites are more frequently dependent of the atrial remodeling and the myocardial substrate (1, 15, 38).

Additional relevant anatomical structures are the ganglionated plexi. In 2004 Pachon et al. described the AF-Nest ablation technique (39). They defined two types of atrial tissue by using spectral analysis of atrial electrograms by applying fast Fourier transformation (39). First, a compact myocardium which shows classical myocardial behavior with fast conduction. Second, a fibrillar myocardium which they compared to a group of nerve cells, characterized by heterogeneous conduction and short refractory periods. They reported clustering of fibrillar myocardium in small areas, which they called AF nests as upon stimulation of these areas ectopy and AF could be induced (39). Later on, they correlated these AF nests with vagal innervation by assessing the response upon extracardiac vagal stimulation before and after ablation of these fibrillar regions (40). In 2007, Lellouche et al. described that a specific pattern of local electrograms, so-called high amplitude fractionated electrograms, prior to ablation was associated with parasympathetic responses defined as an increase in AH-interval with ≥10 ms or a decrease in heart rate ≥20% (41). In retrospect, both Pachon et al. and Lellouche et al. described techniques to determine the endocardial location of the ganglionated plexi.

Lastly, while the ligament of Marshall is the remaining structure after involution of the left superior caval vein, the vein of Marshall remains an active vein with abundant parasympathetic and sympathetic innervation and myocardial bundles (42). Due to its size, the vein of Marshall is targeted using ethanol infusion. While the vein of Marshall has been an origin of triggers and reentrant activity in the AF pathogenesis, it is most commonly targeted to facilitate or complete mitral isthmus block given its posterior epicardial location between the left inferior pulmonary vein and the coronary sinus (42, 43).

## 4 Assessing the autonomic nervous system in atrial fibrillation

The gold standard for assessing cardiac ANS activity is the direct measurement of action potentials, nerve activity, or changes in neurotransmitter concentrations in the sympathetic and parasympathetic nerves (44). The invasiveness of these techniques limits their use in clinical practice. Further, the robustness of measurement of venous and urinary neurotransmitter levels or their spillover is limited due to the rapid clearance of acetylcholine and the fact that circulating noradrenaline represents only a minor fraction of the neurotransmitter secreted from nerve terminals (44, 45). ANS activity is typically assessed by measuring its effect on the innervated organs, therefore ANS response, rather than true activity (46).

### 4.1 Heart rate variability

**Figure 1:**
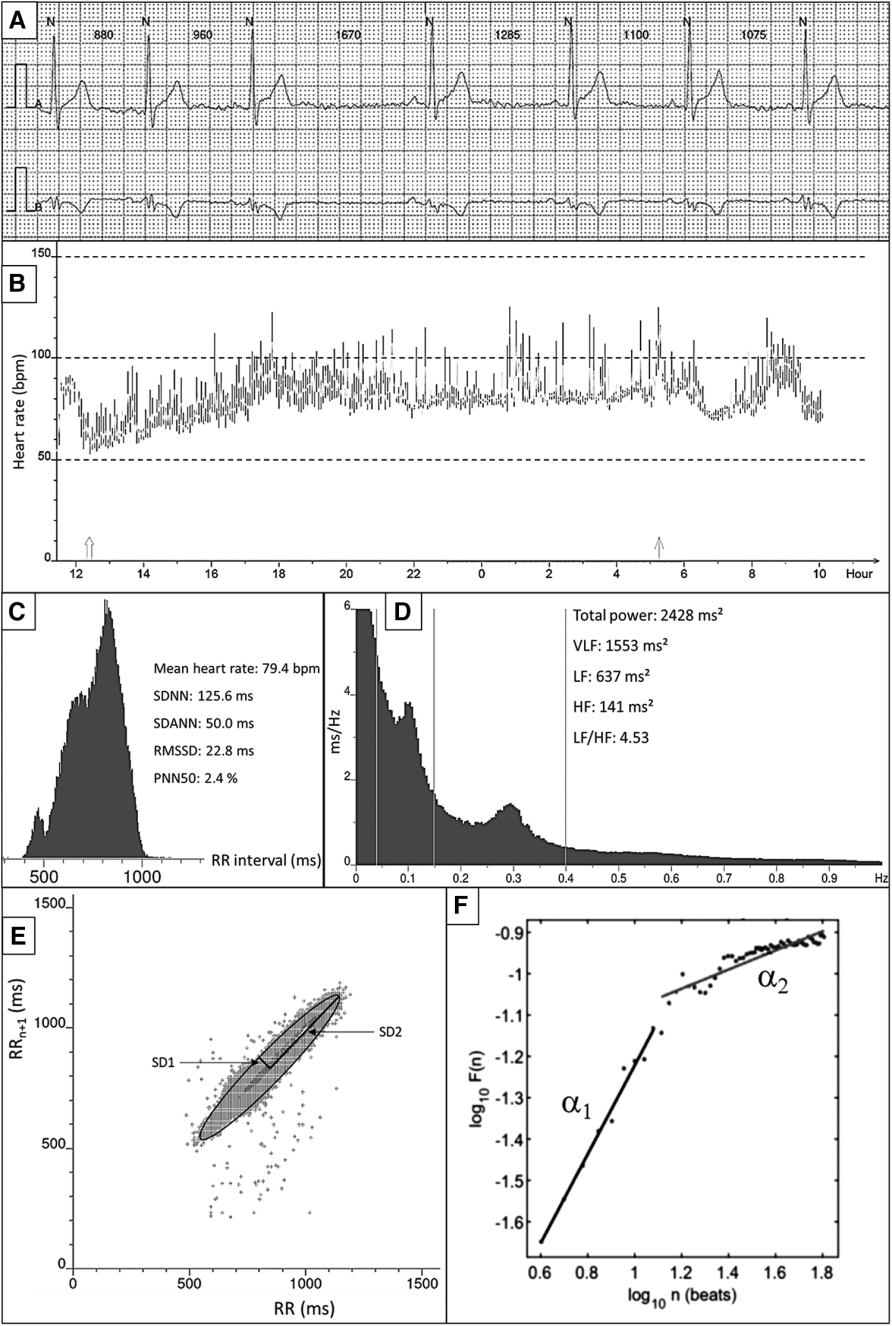
Overview of HRV measurements and techniques. (**A**) Example of raw ECG. All QRS complexes are marked with *N*, identifying normal heartbeats. The RR interval between consecutive beats is shown in milliseconds. (**B**) 24-h tachogram presenting the heart rate on the Y-axis and the time on the X-axis. (**C**) 24-h RR histogram presenting results of the time domain HRV analysis. (**D**) 24 h spectral analysis of the heart rate using a Fast Fourier Transformation with results of the frequency domain HRV analysis. (**E**) Poincaré plot presenting the RR interval between consecutive normal heartbeats. (**F**) Detrended fluctuation analysis.

The most commonly applied methods to assess ANS response is heart rate variability (HRV) (47). These sets of measurements describe the fluctuations in time intervals on a beat-to-beat basis. While a higher HRV is typically considered beneficial, it should be differentiated from pathological conditions where an irregular heart rate mimics a large variability in cycle length, such as AF (48). HRV measures can largely be divided into three groups (Table 1 and Figure 1): time domain, frequency domain, and nonlinear measurements (47). In brief, the time domain indices quantify the variability in cycle length on a beat-to-beat basis, while the frequency domain indices are based on a fast fourier transformation or autoregressive models resulting in the variance of all normal-to-normal beats in predefined frequency bands (47). Nonlinear measurements, such as detrended fluctuation analysis and poincaré plots, assess the complexity, repeatability, and predictability of patterns in the normal-to-normal intervals (47). The standard deviation of normal-to-normal intervals (SDNN) is the standard of time domain measurements and reflects the sum of both sympathetic and parasympathetic activity (49). The root-mean-square of successive differences (RMSSD) is the best time domain measure to assess vagally-mediated changes in HRV and is highly correlated with the high frequency (HF) component in frequency domain analysis. The low frequency (LF) component in frequency domain analysis reflects both sympathetic and parasympathetic nervous system activity. Compared to fast fourier transformation, autoregressive models have several advantages including smoother spectral components, easy post-processing of the spectrum, and lower requirements with regards to the length of the data (50). Further, it should be noted that both short-term and 24-h measurements are reported, where the 24-h measurements are the gold standard and often show a higher predictive power (51).

**Table 1:** Common HRV measures.

	Units	Description
Time domain analysis		
NN interval	ms	Normal-to-normal intervals.
SDNN	ms	Standard deviation of all NN intervals reflecting the sum of symphatic and parasympathetic activity.
SDANN	ms	Standard deviation of the averages of NN intervals in all 5 min segments of the entire recording and correlates with VLF and ULF over a 24 h period.
RMSSD	ms	The square root of the mean of the sum of the squares of differences between adjacent NN intervals reflecting vagally-mediated changes in HRV.
pNN50	%	Percentage of NN intervals differing by more than 50 ms in the entire recording reflecting vagally-mediated changes in HRV.
Frequency domain analysis		
Total power	ms^2^	Variance of all NN intervals
5 min total power	ms^2^	Variance of NN intervals over the temporal segment (Frequency range: ≤0.4 Hz).
ULF	ms^2^	Power in the ultra-low frequency range (≤0.003 Hz)
VLF	ms^2^	Power in very low frequency range (≤0.04 Hz)
LF	ms^2^	Power in low frequency range (0.04–0.15 Hz), reflects both symphatic and (predominantly) parasympathetic activity
LF norm	n.u.	(LF/Total Power-VLF) × 100
HF	ms^2^	Power in high frequency range (0.15–0.4 Hz), reflects parasympathetic activity.
HF norm	n.u.	(HF/Total Power-VLF) × 100
LF/HF		Ratio of LF and HF

Table 2 presents an overview on the findings of relevant studies regarding HRV in AF. Overall, these reports show that persistent AF is associated with lower HRV measures and that HRV shows distinct patterns to differentiate between vagal and adrenergic subtypes (9, 52, 54–56, 58, 62). However, there is an overlap where patients have both distinct vagal and adrenergic onset of AF (9, 56). Typically, HRV is reduced after AF catheter ablation, but patients with AF recurrence either show less pronounced reduction or recovery or HRV measures (53, 57, 59, 60). These associations were also present in a meta-analysis including 16 studies and a total of 2,352 patients, albeit significant publication bias was detected (63).

**Table 2:** Overview of relevant studies describing HRV measures in patients with AF.

First author	Year	*N*	Findings
Van den Berg et al. (52)	1997	28	Baseline HRV was higher in AF group. After administration of methylatropine, HRV neared zero in the control group whereas it returned to baseline in AF group. SD, RMSSD, LF and HF at baseline were significantly (*p* < 0.05) correlated with vagal tone in both groups.
Fioranelli et al. (9)	1999	28	In the 5 min before onset of AF 2 distinct patterns were differentiated based on power spectrum analysis. In half of the episodes there was an increase in sympathetic tone, while the other half had an increase in parasympathetic tone. There was overlap of episodes within subjects.
Akyürek et al. (53)	2003	47	After electrical cardioversion, patients with persistent AF exhibited reduced HRV compared to healthy controls. Patients with AF recurrence had lower HRV compared to those with AF recurrence.
Vikman et al. (54)	2003	78	Increased HF, reflecting enhanced autonomic tone, was associated with AF recurrence after electrical cardioversion of persistent AF. Short-term and non-linear HRV measures were not associated with outcome.
Friedman et al. (55)	2004	38	Reduced HRV correlated with increasing left atrial and left ventricular dimensions. Left atrial dimension was an independent determinant of HRV. HRV greater in “lone AF” than other cardiac disorders.
Lombardi et al. (56)	2004	65	Among 110 paroxysmal AF episodes, approximately 70% were preceded by increase in sympathetic activity while only in 30% a vagal predominance was detected.
Seaborn et al. (57)	2014	83	RF catheter ablation of paroxysmal and persistent AF was associated with reduced HRV. At 1-year follow-up, patients with recurrence had only temporary HRV reduction, while in patients without recurrence the change in HRV was sustained.
Perkiömäki et al. (58)	2014	784	In a middle-aged population, the only HRV predictor in multivariable analysis of new-onset AF was reduced LF.
Vesela et al. (59)	2019	45	There was no difference in change in short-term HRV measures before and after catheter RF ablation when comparing PVI with PVI + GP ablation. Vagal responses during ablation were not different between subgroups.
Marinkovic et al. (60)	2020	100	HRV changes during the 3-month period after RF catheter ablation for paroxysmal AF may predict long-term outcomes. SDNN cut-off value of 62.5 ms showed the best predictive ability for late recurrence AF.
Khan et al. (61)	2021	94	Patients with permanent AF exhibited higher HRV compared to those with paroxysmal AF. However, measurements were obtained in AF.
Kim et al. (62)	2022	782	Higher HRV, assessed by HF, RMSSD, and pNN50, was associated with new-onset AF in patients with hypertension reflecting the importance of increased parasympathetic activity.

### 4.2 Heart rate turbulence

Heart rate turbulence (HRT) describes the perturbation of sinus rhythm cycle length after isolated extrasystoles (64). It provides measurements of the brief acceleration of the heart rate, turbulence onset, and the subsequent gradual deceleration of the heart rate, known as turbulence slope. While HRT is best known for risk stratification of sudden cardiac death where it is based on ventricular extrasystoles, one can also use atrial HRT by using atrial extrasystoles (64–66). Ventricular-based HRT was associated with the incidence and burden of postoperative AF (67). Atrial-based HRT onset has shown clear dynamics before the onset of AF suggesting an important role for transient enhancement of vagal tone (68). When inspecting the graphs, however, one can appreciate that most patients show a significant increase in vagal outflow, but that a smaller subset show the exact opposite suggestive of an adrenergic phenotype. It should be noted, however, that for ventricular-based HRT the site of origin does not influence its results, while for atrial-based HRT the result is highly dependent on the coupling interval and atrioventricular nodal conduction (65, 69). Therefore, instead of truly reflecting autonomic function, the potential of HRT in AF could rather be a surrogate of the pro-arrhythmic potential of short-coupled atrial extrasystoles with delayed AV conduction.

### 4.3 Baroreflex sensitivity

Baroreflex sensitivity (BRS) reflects acute changes in sympathetic-parasympathetic balance during blood pressure variability and is a potent risk stratification tool in cardiovascular diseases (70–72). Baroreceptors located on the aortic and carotid wall sense acute changes in blood pressure and counteract with changes in heart rate and systemic vascular resistance. As the acute changes in cardiac sympathetic activity are too slow to respond to beat-to-beat changes in blood pressure, BRS is considered to reflect vagal activity. BRS is expressed as the change in interbeat intervals in milliseconds per unit change in blood pressure (70). Higher values indicate more pronounced vagal activity. However, it should be noted that BRS does not represent the blood pressure buffering capacity but only measures the reflex effect on the sinus node, and BRS does not take into account the direction of the effects (increase or decrease). Acute increases in BRS preceding onset of AF has been described, suggesting a typical vagally-mediated AF subtype (73). Further, BRS was severely impaired during AF, but could be restored by maintaining rhythm control (74). Before catheter ablation, but measured during sinus rhythm, patients with persistent AF had a significant lower BRS when compared to those with paroxysmal AF (75).

Data on BRS after catheter ablation is limited. Both Kondo et al. and Miyoshi et al. described a significant reduction in BRS after radiofrequency catheter ablation (75, 76). Further, both described a significant difference in change between patients with AF recurrence and those without AF recurrence (75, 76). In the study by Kondo et al. the reduction in BRS was 5.0 ± 4.0 ms/mmHg in patients without recurrence, which was significantly larger than the 1.9 ± 1.4 ms/mmHg observed in patients with AF recurrence (*p* = 0.019) (76). Miyoshi et al. observed a slightly lower BRS in patients with persistent AF when compared to paroxysmal AF [2.97 (IQR 0.52–6.62) vs. 4.70 (IQR 2.36–8.37) ms/mmHg, *p* = 0.047] (75). Further, the reduction in BRS was more pronounced in patients with paroxysmal AF when compared to those with persistent AF. The reduction in BRS was significantly smaller in paroxysmal AF patients with recurrence, when compared to those without recurrence [4.21 (IQR2.50–8.19) vs. 1.97 (IQR 0.46–2.88) ms/mmHg, *p* = 0.011] (75). The latter was not present in patients with persistent AF and recurrence.

### 4.4 Other

The concept of P-wave alternans or atrial alternans is currently under investigation in the setting of AF (77). Similar to the well-known albeit nearly abandoned T-wave alternans, atrial repolarization dynamics reflect sympathetic nervous system activity by measuring beat-to-beat differences in action potential dynamics due to changes in cytosolic calcium levels (77). Distinct differences in action potential restitution curves have been observed when comparing vagally-mediated and adrenergic AF models (78). Hence, atrial alternans could be of interest to differentiate these AF subtypes, but most contemporary data is based on in-silico or animal models (77). This is partly due to the challenging technical requirements to measure atrial alternans.

Alternative measures of ANS activity, such as post-extrasystolic potentiation, self similarity, deceleration capacity, salivary gland activity, and sudomotor function, have been poorly or not studied in the setting of AF (79, 80).

## 5 Discussion

Over time, the role of the autonomic nervous system and its role in modulating AF pathophysiology has been consistently documented. Surges of both sympathetic and parasympathetic activity have been associated with the onset of AF. Differentiating these vagal and adrenergic AF paroxysms is however challenging as patients may exhibit both subtypes. How this knowledge can be applied to improve AF catheter ablation outcomes requires further investigation. Based on previous randomized clinical trials, some patients might benefit from autonomic denervation. Given the wide range of therapeutic interventions, the question is how can we identify which patients will best benefit from a certain intervention and evolve towards personalized AF treatment strategies. Identifying patients who might benefit from parasympathetic denervation may be challenging due to the overlap phenomenon. Also, rather than detecting AF current research is focusing on prediction of AF burden, while patients with vagal subtypes may have less frequent episodes when linked to specific triggers (81–83). Novel artificial intelligence algorithms based on HRV features have high accuracy in predicting AF based on the ECG signal just a few minutes before AF onset (82, 83). Hence, in the era of digital health and wearables there may be an opportunity to collect data and improve our understanding on how we can differentiate vagal and adrenergic AF subtypes and guide treatment strategies in future randomized clinical trials.

## 6 Conclusion

While the existence of a vagal AF subtype cannot be denied, there is no universal definition of a vagal AF. This is mainly due to the fact that vagal and adrenergic AF subtypes represent the extremes of a spectrum where most of the patients will present with a mixed subtype. Therefore, it is important to note that the described characteristics are not definitive markers for either subtype or that there may be significant overlap. Differentiation of vagal and adrenergic AF subtypes may have implications for the treatment options as certain interventions have shown not to modify the autonomic innervation of the heart. Non-invasive methods for identifying patients with vagally-mediated AF, such as ambulatory HRV, may have potential to guide ablation strategy. However, further research is needed to better understand the mechanisms underlying the relationship between autonomic tone and AF, and to validate the use of measures such as HRV, baroreflex sensitivity, or novel artificial intelligence algorithms, as tool for patient selection.
